# Variable- and person-centered approaches to examining construct-relevant multidimensionality in writing self-efficacy

**DOI:** 10.3389/fpsyg.2023.1091894

**Published:** 2023-02-20

**Authors:** Morgan Les DeBusk-Lane, Sharon Zumbrunn, Christine Lee Bae, Michael D. Broda, Roger Bruning, Ashlee L. Sjogren

**Affiliations:** ^1^Department of Foundations of Education, School of Education, Virginia Commonwealth University, Richmond, VA, United States; ^2^College of Education and Human Sciences, University of Nebraska–Lincoln, Lincoln, NE, United States; ^3^Youth-Nex Research Center, The School of Education and Human Development, University of Virginia, Charlottesville, VA, United States

**Keywords:** writing, self-efficacy, latent profile analysis, bifactor, latent model

## Abstract

Self-efficacy is an essential component of students’ motivation and success in writing. There have been great advancements in our theoretical understanding of writing self-efficacy over the past 40 years; however, there is a gap in how we empirically model the multidimensionality of writing self-efficacy. The purpose of the present study was to examine the multidimensionality of writing self-efficacy, and present validity evidence for the adapted Self-Efficacy for Writing Scale (SEWS) through a series of measurement model comparisons and person-centered approaches. Using a sample of 1,466 8th–10th graders, results showed that a bifactor exploratory structural equation model best represented the data, demonstrating that the SEWS exhibits both construct-relevant multidimensionality and the presence of a global theme. Using factor scores derived from this model, we conducted latent profile analysis to further establish validity of the measurement model and examine how students disaggregate into groups based on their response trends of the SEWS. Three profiles emerged, differentiated by global writing self-efficacy, with substantively varying factor differences among the profiles. Concurrent, divergent, and discriminant validity evidence was established through a series of analyses that assessed predictors and outcomes of the profiles (e.g., demographics, standardized writing assessments, and grades). Theoretical and practical implications and avenues for future research are discussed.

## Introduction

“Self-belief does not necessarily ensure success, but self-disbelief assuredly spawns failure”

-Bandura, *Self-Efficacy: The Exercise of Control, 1997*

As a foundational component to Bandura’s (1997) social cognitive theory, self-efficacy, or “beliefs in one’s capabilities to organize and execute the courses of action required to produce given attainments” (p. 3), is an integral component to the function of human agency. Self-efficacy describes how self-perceptions of capacity to perform tasks and skills influence one’s behavior, affect, persistence, and achievement. The act of writing necessitates various interrelated sub-skills, frames, and procedures – spelling, grammar usage, punctuation, organization, voice, prose – and the ability to orchestrate them in a cohesive manner. As a highly complex and challenging process, writing self-efficacy plays an important role in writing success (Pajares and Johnson, 1996; Graham et al., 2019; Wijekumar et al., 2019). And, given that writing is instrumental in society, research over the past several decades has focused great attention to how, why, and to what degree efficacious beliefs influence writing performance and the relationship between writing self-efficacy and other forms of motivation (see Klassen and Usher, 2010; Honicke and Broadbent, 2016). However, little attention has focused on examining psychometrically sound instruments to capture the multidimensionality of writing self-efficacy (Zumbrunn et al., 2020). The overarching purpose of this study was to examine the dimensionality of writing self-efficacy and build validity evidence for a measure of writing self-efficacy – the adapted Self-Efficacy for Writing Scale (SEWS; Ekholm et al., 2015; Zumbrunn et al., 2016).

### Writing self-efficacy

Effective writing requires more than adequate knowledge, skills, and competencies. As is true of performance across every academic domain, successful writing also requires efficacy beliefs strong enough to regulate knowledge, skills, and competencies throughout the writing process (Bandura, 1993). Integral to both effort and persistence (Bandura, 1997; Schunk and Usher, 2012; Schunk and DiBenedetto, 2016), self-efficacy has been extensively studied as a major component to writing motivation (Pajares, 2003, 2007; Schunk, 2003). For example, prior studies illustrate writing self-efficacy’s relation to powerful motivational concepts such perceived value (Shell et al., 1995), self-concept (Pajares et al., 2000), attitudes (Bruning et al., 2013), and apprehension (Sanders-Reio et al., 2014). Writing self-efficacy has shown to be a robust predictor of writing achievement across many studies, making a strong and independent contribution to writing performance, even when controlling for prior ability or achievement (Shell et al., 1989, 1995; Zimmerman and Bandura, 1994; Pajares and Johnson, 1996; Pajares and Valiante, 1997, 2001; Pajares et al., 1999, 2007a; Bruning and Horn, 2000; Troia and Graham, 2016, Graham et al., 2017, 2019; Wijekumar et al., 2019). Furthermore, Graham’s (2018) Writer(s)-Within-Community model suggests beliefs associated with writing capabilities are a core component to how writers situate themselves within specific contexts, times, places, environments, or writing communities, while also contributing to the “capital” they bring forward. In doing so, such beliefs are influential not only to the production of writing (see Hayes, 1996), but also important to the moderating influence of control mechanisms (e.g., decisions, attention regulation, agency, emotions, and thoughts).

Though the depth of literature on writing self-efficacy underscores the value in understanding, measuring, and optimizing student writing self-efficacy, the ability to capture, measure, or otherwise operationalize writing self-efficacy has not been without difficulty (Pajares, 2003; Klassen and Usher, 2010). Self-efficacy researchers have consistently been warned that without adhering to proper item wording, time-vantage, focus, and conceptualization, “the future of self-efficacy research as a theoretically grounded means of understanding human behavior is threatened” (Klassen and Usher, 2010, p. 20). Given this, the field has recently progressed both in its theoretical alignment and focus on specific process-oriented facets within the domain of writing (Klassen and Usher, 2010; Bruning et al., 2013). However, little research has focused on the psychometric properties of measures of writing self-efficacy (Tate and Warschauer, 2018).

#### The measurement and structure of writing self-efficacy

Over the past 40 years, researchers have used various methods of item reduction [e.g., exploratory factor analysis (EFA)], reliability, and confirmatory factor analyses (CFAs), to evaluate the psychometric quality of writing self-efficacy measures (McCarthy et al., 1985; Shell et al., 1989, 1995; Pajares et al., 1999; Pajares and Valiante, 2001). Contemporary work increasingly applies advanced psychometric methods (Brown, 2015; Kline, 2016). For example, work by Engelhard and Behizadeh (2012) used Rasch measurement theory (a type of item response theory; Rasch, 1960) to examine the psychometric quality of the Writing Self-Efficacy Scale (WSES; Pajares et al., 1999). Similarly, De Smedt et al. (2017, 2018) and Zumbrunn et al. (2020) employed structural equation models to examine writing self-efficacy’s relationship to other motivational and cognitive constructs.

Writing self-efficacy has been commonly depicted as a unidimensional factor (Pajares and Valiante, 2006); however, a growing literature suggests that it is multidimensional (Bruning et al., 2013; MacArthur et al., 2015; De Smedt et al., 2016, 2017, 2018; Zumbrunn et al., 2020). This newer research has consistently added and organized items focused on efficacy toward writing self-regulation (e.g., focus, strategy use, and planning) and other cognitive components (e.g., ideation, creativity, and idea development) involved in the writing process (e.g., Bruning et al., 2013; MacArthur et al., 2015; Graham et al., 2017, 2019; Wijekumar et al., 2019). Of these, Bruning et al.’s (2013) SEWS focuses on the efficacious beliefs of *ideation*, traditional writing *conventions*, and *self-regulation*, and has been widely used and adapted since publication (e.g., Ekholm et al., 2015; De Smedt et al., 2016, 2017, 2018; Zumbrunn et al., 2016; Ramos-Villagrasa et al., 2018). Therein, *ideation* serves to depict a writer’s efficacy beliefs of their ability to produce, create, and use ideas. *Conventions*, like many measures focused on writing’s skills and tasks, seeks to capture a writer’s efficacy beliefs associated with common standards, such as grammar and spelling, that are employed to communicate with writing. Lastly, *self-regulation* depicts a writer’s confidence to “direct themselves” (affective response), organize, and navigate through the writing process (Bruning et al., 2013).

Several studies have confirmed the multidimensional factor structure originally portrayed by Bruning et al. (2013), De Smedt et al. (2016, 2017, 2018), and Yilmaz Soylu et al. (2017). Additional studies have adapted or extended the SEWS to new languages and samples (Ekholm et al., 2015; Zumbrunn et al., 2016; Ramos-Villagrasa et al., 2018). Ekholm et al. (2015) adapted the SEWS by reducing it to 9 items, yet in doing so confirmed a single factor structure with an undergraduate sample. Extending this work to be more developmentally appropriate for younger writers, Zumbrunn et al. (2016) further adapted the SEWS by adjusting the traditional 0–100 rating scale to a 0–4 rating scale. Incorporating both adaptations, recent work by Zumbrunn et al. (2020), which used a 9-item, 0–4 rating scale, adaptation of the SEWS, found a 3-factor measurement structure invariant across elementary and high-school students. Furthermore, DeBusk-Lane et al. (2021) and Zumbrunn et al. (2020) found a 3-factor measurement structure of the adapted SEWS with both elementary and secondary school students. Although a well-fitting 3-factor structure is seemingly evident across developmental spectrums, this structure has also exhibited statistical clues (e.g., strong latent factor correlations) that suggest other models may more accurately model the data. This study will extend the existing literature by testing CFA, exploratory structural equation models (ESEM), and bifactor ESEM models that consider various perspectives of modeling factor relationships and the potential presence of a global factor.

#### Aligning a measurement model with theory

Two issues have emerged related to the ways in which the SEWS has traditionally been modeled. First, because the measure was originally constructed to capture efficacious beliefs of writing collectively through multiple related dimensions, it is likely that it does, in fact, represent both global and specific constructs. It is both theoretically aligned and logically plausible to expect subscales within a measure with related domain-specific facets to exhibit some amount of a global (or hierarchical) factor that reflects participants’ overall sense of writing self-efficacy (Reise et al., 2013). Theoretically, Bandura (1997) explained that self-efficacy factors may share similar subskills, incorporate skills that are developed together, enact similar self-regulatory mechanisms, use similar approaches to problem solving, and query constructs that similarly draw from past experiences that have bolstered one’s belief in their ability, thus implying a multidimensional factor structure.

Further, recent empirical evidence brings into question whether the adapted SEWS is best modeled by three distinct factors (Zumbrunn et al., 2020, 2021; DeBusk-Lane et al., 2021) or a single factor alone (Ekholm et al., 2015; Zumbrunn et al., 2016). Across both the original 16- and the adapted 9-item measures, moderate latent factor correlations, large first factor eigenvalues, and moderate correlations among the specific latent factors to other unidimensional writing self-efficacy measures suggest the presence of a hierarchical or global factor (e.g., Reise et al., 2013; MacArthur et al., 2015; Ramos-Villagrasa et al., 2018; Zumbrunn et al., 2020, 2021; DeBusk-Lane et al., 2021).

Second, it can be expected that efficacious beliefs derived and exhibited by items that query beliefs associated with “writing even when it is difficult” likely translate and extend to cross-factor items that query beliefs associated with a writer’s effort to “think of words to describe my ideas.” This conceptual overlap suggests that items may be related to more than one specific factor. Therefore, because the items themselves are imperfect indicators that likely associate with other similar latent constructs, aside from their *a priori* forced factor relationship, current depictions through CFA may not fully depict reality (Asparouhov et al., 2015; Morin et al., 2016a, 2017).

Together, these two hypothesized influences (i.e., global or hierarchical factor and item cross-factor relationships or cross-loadings) are referred to as sources of construct-relevant psychometric multidimensionality (Morin et al., 2016b, 2017). In typical CFA models, item factor relationships restrict cross-loadings to zero, forcing true-score variability between factors (of both cross-loading and hierarchical/global factors) to be absorbed by only *a priori* factors, negating both the presence of hierarchically ordered and conceptually overlapped constructs, which may result in bias parameter estimates (Asparouhov et al., 2015).

Given these issues, there is a clear need to further examine how the SEWS’ is modeled. To further examine the presence of a global construct, various bifactor or hierarchical models may more accurately model efficacy beliefs derived collectively from the SEWS’ measurement items. Additionally, to better understand how the items interrelate, measurement models that allow multiple cross-loadings between items and multiple latent factors (e.g., ESEM) may provide a better vantage of the unique relationships between conceptually related items.

Beyond gaining a better understanding of how to best model the SEWS, there is also ample room to explore the measure’s validity. In this case, although the original SEWS has been related to other psychological and motivational constructs (see De Smedt et al., 2017, 2018; Zumbrunn et al., 2020), these constructs are commonly modeled by either composite scores (specific factor item means) or latent factor values in variable-centered analyses. Variable-centered approaches rely on the assumption that all participant data are collected from a uniform population from which averages are derived, whereas person-centered approaches assume the sample may include several sub-populations (Masyn, 2017). Specifically, variable-centered approaches (factor models) “decompose” covariances to describe relationships between and among variables, while person-centered approaches use covariances to explain and describe relationships between individuals (Bauer and Curran, 2004). The person-centered approach taken in this study allowed us to examine the possibility that students may not be uniform across all dimensions of writing self-efficacy, but rather, that there are subgroups of students characterized by unique clusters of writing self-efficacy dimensions. Although there are many person-centered approaches (e.g., hierarchical clustering, and *K*-means), we used latent profile analysis (LPA). Comparatively, LPA is a model-*based* approach that provides a probability-based classification generated from maximum likelihood methods, misclassification (error) estimates, more nuanced group membership mean estimates, various fit statistics to help determine the number of groups, and classification error adjusted analyses related to group predictors and outcomes (Magidson and Vermunt, 2002).

The purpose of this study was to examine the multidimensionality of writing self-efficacy using ratings from the adapted SEWS and provide further validity evidence (Ekholm et al., 2015; Zumbrunn et al., 2016). To date, no other study has examined the adapted SEWS beyond traditional CFA model depictions, which have been shown to be limited and less accurate among multidimensional measures (Morin et al., 2016a, 2017). With the growing trend of statistically assessing latent concepts with structural equation modeling, it is important to accurately model the data to ensure relational parameter estimates represent true scores and construct-irrelevant variation. To better understand and help further validate the SEWS, this study will employ LPA to identify unique clusters of writing self-efficacy, as well as continue to examine predictors (e.g., demographics) and related outcomes (e.g., standardized writing assessments) of the identified profiles. To demonstrate validity evidence, the adapted SEWS will also be examined as it relates to both writing apprehension and a separate writing self-efficacy measure, the WSES (Pajares, 2007).

## Materials and methods

This work is guided by a series of research questions that first assess the presence of two sources of construct-relevant multidimensionality (RQ1 and RQ2), and then examine the dimensionality and profile validity using a person-centered approach (RQ3 and RQ4).

1. Are the items of the SEWS conceptually related across *a priori* factors?
2. Does the SEWS exhibit hierarchically ordered constructs?
3. What specific quantitative profiles of writing self-efficacy emerge?
4. What forms of validity evidence are found for the profiles of the SEWS?
  a. Do the profiles exhibit concurrent validity evidence based on responses to the WSES?
  b. Do the profiles exhibit divergent/discriminant validity evidence based on responses to the Writing Apprehension Scale (WAS-12)?
  c. Do the profiles exhibit predictive validity?

### Participants

All 1,466 participants were 8th through 10th graders in a large southeastern school division in the United States. During 2018–2019 school year, this division consisted of 48.5% female, and 32.0% identified as economically disadvantaged [which includes those eligible for Free/Reduced Meal or receives Temporary Assistance for Needy Families (TANF), those eligible for Medicaid, or Identified as either migrant or experiencing Homelessness], 9.8% English Language Learners (ELL), and 12.5% students with disabilities. The division is also racially diverse, including students who identify as less than 1% American Indian or Alaskan Native, 3.3% Asian, 25.6% Black or African American, 49.3% White, 16.4% Hispanic, less than 1% Native Hawaiian or Other Pacific Islander, and those who identified as non-Hispanic, but two or more races 4.9%. Demographics across grades 8 through 10 are comparable to the overall averages.

### Measures

#### Demographic variables

To both accurately describe the sample and provide validity evidence of profiles, we requested several demographic and prior performance measures from the partnering school division, including participants’ sex, race/ethnicity, first quarter grades, and standardized writing scores.

#### Writing self-efficacy

The adapted SEWS (Ekholm et al., 2015; Zumbrunn et al., 2016), originally developed by Bruning et al. (2013), was the primary measure for this study. The modified version of this scale consists of nine items that ask students to rate, on a scale form 1 (*Almost never*) to 4 (*Almost always*), how confident they are that they can perform specific writing processes. Two studies reported McDonald’s Omega (Deng and Chan, 2017; McNeish, 2017) for each factor: conventions, ideation, and self-regulation at 0.65, 0.79, and 0.80, and 0.61, 0.77, and 0.75, respectively (Zumbrunn et al., 2020; DeBusk-Lane et al., 2021). The full scale is provided in the Supplementary material.

#### Validity-building predictors and outcomes

To support a substantive interpretation and develop validity evidence of the profiles, the person-centered approach used several predictors and outcomes. In addition to assessing the demographic variables, we also examined two other measures to provide additional criterion-related validity evidence: the WSES (Pajares, 2007) and a shortened version of the WAS (Daly and Miller, 1975; Pajares and Johnson, 1994; Bline et al., 2001), the 12-item Writing Apprehension Scale (WAS-12; Limpo, 2018). The WSES was chosen, based on both its broad usage in prior literature and the extent to which it has been statistically evaluated, to provide concurrent validity evidence to the SEWS. The WAS-12 was chosen, also based on its extensive use and statistical reliability, to provide concurrent divergent/discriminant validity evidence. Lastly, a standardized writing assessment across the grades was examined as a primary outcome.

*Standardized writing assessment scores* (8th and 10th grade). Both the 8th and 10th grade participants participated in a statewide standardized writing assessment. For all students, the first component required students to correct errors embedded in sections of a notional rough draft of student writing. The second component required students to write a short paper in response to an expository or persuasive prompt; papers were scored on a scale of 1 (low) to 4 (high) by two trained readers using a holistic rubric including the components of composing/written expression and usage/mechanics. At the time of this study, the school division was piloting a new performance-based writing assessment that required a local rubic – no computation of reliability is available. Documentation that guided the development of the grading rubric may be found in Supplementary material. This assessment was conducted approximately 2-weeks after participants completed all other measures included in this study. Therefore, this assessment served to provide predictive validity by inspecting the relationship between writing efficacy beliefs and writing performance.

*Writing Self-Efficacy Scale* (Pajares, 2007). The WSES scale consists of 10 items asking students how sure they are at performing a specific writing skill on a scale of 0 (*no chance*) to 100 (*completely certain*). Pajares (2007) reported a two-factor solution representing basic grammar skills and advanced composition skills, individual factor Cronbach alpha coefficients of 0.88 and 0.86 respectively, and similar factor and reliability findings at the elementary, middle school, and high-school levels, among 1,258 students from grades 4–11.

*Writing Apprehension Scale-12* (Limpo, 2018). The WAS-12 is a 12-item shortened version of the 63-item WAS originally presented by Daly and Miller (1975) that was, through item reduction, reduced to 26 items representing a single factor. Similarly, through item reduction techniques, 12 items that represented two salient factors, concern and affect, were presented with Cronbach’s alphas for each facet greater than 0.85 (Limpo, 2018).

Importantly, the WAS-12 was previously presented with concurrent validity to Pajares and Valiante (1999) WSES, where the “affect” (*I like writing*) facet was positively correlated (although not significantly) and the “concern” facet was negatively significantly related. These findings are in-line with previous work that has examined writing anxiety and writing self-efficacy (Pajares and Johnson, 1994; Pajares and Valiante, 1999; Goodman and Cirka, 2009; Martinez et al., 2011; Sanders-Reio et al., 2014; Limpo, 2018).

### Procedures

All survey data was collected in January 2018 as part of the partnering school division’s priority to assess student writing motivation. Survey data was collected online, and each item was presented iteratively with the overall directions for each applicable section as a header. Students had no time limit to complete the survey, and teachers were instructed to not provide help in clarifying or explaining survey directions or items. All psychological measures were collected in one sitting in each student’s English class.

### Analysis

The data analytic plan encompassed two phases, a variable-centered approach that consisted of multiple factor model comparisons, and a person-centered approach that consisted of a LPA and subsequent analyses.

#### Variable centered analyses (RQ1 and RQ2)

**FIGURE 1:**
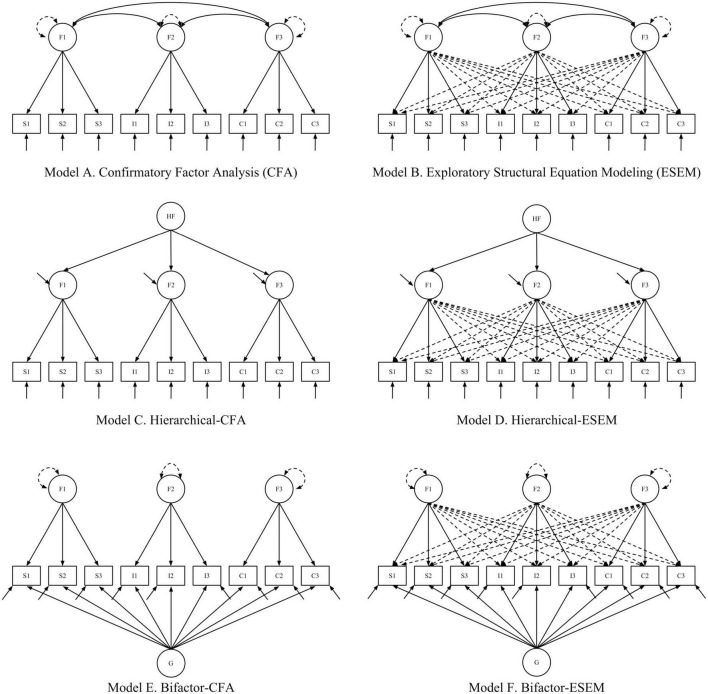
Measurement models based on the 3-factor Self-Efficacy for Writing Scale (SEWS).

The analyses, unless otherwise noted, were estimated in Mplus version 8.2 using the robust weighted least square estimator using diagonal weight matrices for the factor models (WLSMV; Muthén and Muthén, 1998–2017). All measurement models are depicted in Figure 1.

To answer RQ1 and RQ2, which focus on examining the SEWS’ hierarchical and item cross-association, several model comparisons were needed. In total, participant responses on the SEWS were represented with seven models: EFA, CFA, hierarchical CFA (h-CFA), bifactor CFA (b-CFA), ESEM, hierarchical-ESEM (h-ESEM), and a bifactor-ESEM model (b-ESEM). For all models, we report item descriptive statistics [distribution, polychoric correlation coefficients (Finney and DiStefano, 2006), model-based omega coefficients of composite reliability (Deng and Chan, 2017; McNeish, 2017)], standardized factor loadings, and model fit indices. When applicable, we report omega hierarchical or omega hierarchical subscale coefficients to extract how much variability accounted for by the global factor (Rodriguez et al., 2016).

#### Model evaluations

Model evaluations in this study relied on goodness-of-fit indices and the substantive interpretation of parameter estimates, as the use of the Chi-square test of exact fit and the Chi-square differences test is biased due to sample size and model misspecifications (Marsh et al., 2005; Kline, 2016). We used the following: the comparative fit index (CFI; Bentler, 1990); the Tucker–Lewis index (TLI; Tucker and Lewis, 1973); the root-mean-square error of approximation (RMSEA; Steiger, 1990; and its 90% confidence interval); and the standardized root-mean-squared residual (SRMR; Asparouhov and Muthén, 2018). Following established guidelines (e.g., Marsh et al., 2005; Kline, 2016), CFI and TLI greater than 0.9 and 0.95 was considered indicative of excellent fit to the data, respectively. For RMSEA and SRMR, values less than 0.05 and 0.08 are contended to be of excellent fit to the data, respectively (Hu and Bentler, 1998, 1999; Asparouhov and Muthén, 2009, 2018). Additionally, each model comparison included inspections of parameter estimates, statistical conformity, and theoretical adequacy (Fan and Sivo, 2009).

As suggested in Morin et al. (2016a), the CFA and ESEM model was first compared (RQ1). Assuming the ESEM target factor loadings remain strong and well-established (similar to CFA), the precision for which the factor correlations are modeled will likely be superior in the ESEM and reduced (Asparouhov et al., 2015). Unexpected and theoretically difficult to explain cross-loading in the ESEM model could suggest needed changes at the item level. Next, depending on which initial model fit the data best (CFA vs. ESEM), its corresponding hierarchical and bifactor model was compared (RQ2). To be clear, subsequent model comparisons for RQ2 were directly dependent on the optimal model from RQ1.

#### Person-centered analyses (RQ3)

We used factor scores derived from the best fitting variable-centered measurement model in the person-centered approach. Factor scores were derived from Mplus (Muthén and Muthén, 1998–2017). This process will model qualitative differences between profiles over and above any globally held attribute of writing self-efficacy, while also providing clarity of Global (G)-factor differences between profiles.

Using this approach, we extracted profiles using Mplus 8.2’s (Muthén and Muthén, 1998–2017) MLR estimator, 10,000 random starts, 1,000 iterations for the random starts, and 500 final stage optimizations (Hipp and Bauer, 2006). To generate iterative profiles of increasing profiles, we used MplusAutomation, which is an R package used to systematically execute several Mplus input files, to arrange and run all enumeration files (Hallquist and Wiley, 2018; R Core Team, 2019).

For enumeration, we estimated LPAs with 2–7 profiles using the factor scores (Nylund-Gibson and Masyn, 2016) derived from the best fitting measurement model. Following the split-sample cross-validation procedures outlined in Masyn (2013), we randomly split (stratified) the sample approximately equally into “calibration” and “validation” sets, representative to sex, ELL, and grade level (other covariates were not representative to this split due to sample size considerations). Once split, the following enumeration process was performed on the calibration data.

To enumerate these data, we selected models based on multiple statistical indices, theoretical interpretability, and substantive meaningfulness (Nylund et al., 2007; Marsh et al., 2009). Statistical indices included minimum values of Akaike information criterion (AIC), Bayesian information criterion (BIC), and sample-size adjusted BIC (aBIC). Smaller values of AIC, BIC, and aBIC estimates indicate more parsimony when comparing models (Collins and Lanza, 2010; Geiser, 2013). The entropy value and classification probabilities were also examined, with values closer to 1 indicating higher precision and reliability of classification (Jung and Wickrama, 2008). Although entropy alone was not used as a determinant metric, it offers valuable information about how the profiles relate and are distributed (Lubke and Muthén, 2007). We also employed the bootstrapped likelihood ratio test (BLRT), and the Vuong-Lo-Mendell-Rubin likelihood ratio test (VLMR-LRT) to compare nested models (Muthén and Asparouhov, 2012). These model comparison tests compare the model with *k* latent classes to the model with *k−*1 latent classes, whereby a non-significant *p*-value indicates the *k−*1 class should be favored (Muthén and Asparouhov, 2012). It should be noted that these indices and tests are heavily influenced by sample size (Marsh et al., 2009). In such cases, these indices will continually suggest an increasing number of profiles, as AIC and BIC will continue to decline as profiles increase, suggesting each is a better fitting model. To mitigate this, we used elbow-plots to graphically depict information criteria, where the point after the slope flattens is recommended as the optimal number of profiles (Petras and Masyn, 2010; Morin et al., 2011). All enumeration statistics are reported in Supplementary material.

Once a profile solution was determined from the calibration data, we followed the split-sample double cross-validation procedures outlined in Masyn (2013). If successful, the model would be used for the entire sample (Collins et al., 2010; Masyn, 2013). If unsuccessful, a more substantive approach would be taken, whereby similar profiles found between the calibration and validation data would be assessed for similarity (Morin et al., 2016d). Like common measurement model invariance testing, Morin et al. (2016d) procedures compare models across increasing equality constraints to assess configural, structural, dispersion, and distributional similarity.

*Predictor analyses (RQ4a and RQ4b).* We assessed each predictor [sex, race/ethnicity, grade, and prior year standardized assessment (when applicable)] for its influence on profile membership both individually and collectively. Scores from both the WSES and the WAS-12 were also included as predictors to add validity evidence to the profiles. We used Mplus’ R3STEP procedure (to account for profile classification error) that results in a series of multinomial logistic regressions to examine how each predictor alone, and accounting for the others, influenced the likelihood of membership in the profiles.

*Outcome analyses (RQ4c).* Each outcome (WSES, WAS-12, and standardized writing assessments) was assessed across the profiles. Using a similar statistical approach as R3STEP, Mplus’ BCH method evaluates the means of outcome variables across profiles (Vermunt, 2010; Bakk and Vermunt, 2016).

## Results

### Descriptive statistics

Table 1 displays all disaggregated demographic data for sex, race/ethnicity, and grade level for the total sample of students. Minoritized race/ethnicity groups included students from the following backgrounds: American Indian or Alaskan Native, Black or African American, Hispanic, Native Hawaiian or Other Pacific Islander, and those who identified as non-Hispanic, but two or more races.

**TABLE 1:** Descriptive statistics for demographic variables.

	*N*%		Sex				Minority			
			Male		Female		Non-minority		Minority	
*N*%	1,466		727	0.50	739	0.50	810	0.55	656	0.45
**Grade**										
8	203	0.14	117	0.08	86	0.06	152	0.10	51	0.03
9	488	0.33	213	0.15	275	0.19	252	0.17	236	0.16
10	775	0.53	397	0.27	378	0.26	406	0.28	369	0.25

Overall, item response distributions were commonly negatively skewed, yet still within normally accepted ranges of −1 to 1 (Kline, 2016; Table 2). The “conventions factor,” however, was noticeably negatively skewed (Item 1 = −2.277; see Table 2) and exhibited strong kurtosis. Omega values for the SEWS’ original 3-factor structure were adequate (ω = 0.58–0.76) and similar to past work reporting omega composite reliability (Zumbrunn et al., 2020).

**TABLE 2:** Adapted Self-Efficacy for Writing Scale response frequencies and descriptive statistics.

	*N*	Almost never (1)		2		3		Almost always (4)		*M*	σ ^2^	Skewness	Kurtosis
**Self-efficacy for ideation**		***n***	***p***	***n***	***p***	***n***	***p***	***n***	***p***				
ω = 0.79, CI [0.763, 0.805]													
2. I can think of many words to describe my ideas.	1,466	27	0.018	199	0.136	691	0.471	549	0.374	3.216	0.241	−0.628	−0.039
6. I can think of many ideas for my writing.	1,466	79	0.054	313	0.214	630	0.430	444	0.303	2.994	0.721	−0.482	−0.465
7. I can put my ideas into writing.	1,466	46	0.031	252	0.172	619	0.422	549	0.374	3.149	0.650	−0.629	−0.276
Self-efficacy for mechanics													
ω = 0.62, CI [0.582, 0.658]													
1. I can write complete sentences.	1,466	4	0.003	41	0.028	245	0.167	1176	0.802	3.776	0.241	−2.277	5.253
3. I can punctuate my sentences correctly.	1,466	21	0.014	158	0.108	580	0.396	707	0.482	3.359	0.513	−0.857	0.164
5. I can spell my words correctly.	1,466	44	0.030	190	0.130	609	0.415	623	0.425	3.239	0.623	−0.809	0.085
Self-efficacy for self-regulation													
ω = 0.78, CI [0.762, 0.802]													
4. I can concentrate on my writing for a long time.	1,466	116	0.079	446	0.304	603	0.411	301	0.205	2.742	0.761	−0.196	−0.682
8. I can avoid distractions when I write.	1,466	235	0.160	484	0.330	545	0.372	202	0.138	2.485	0.832	−0.045	−0.811
9. I can keep writing even when it is difficult.	1,466	186	0.127	523	0.357	548	0.374	209	0.143	2.541	0.774	−0.031	−0.710

Omega coefficients of composite reliability were computed using 1,000 bootstrapped samples along with bias corrected confidence intervals (see Zhang and Yuan, 2016). By scale response, both the sub-sample quantity (*n*) and the proportion ($\hat{p})$ are provided.

### Variable-centered findings

The EFA models suggested the presence of three salient factors, aligned with *a priori* item-to-factor loadings with adequate fit (see Table 3). All confirmatory and ESEM models provided adequate fit to the data (CFI: 0.981–1.000, TLI: 0.971–1.000; see Table 3), however, as the models progressed, they generally continued to improve. An exception, the h-CFA’s fit declined compared to the base 3-factor CFA. Judging from these fit statistics alone, the bifactor ESEM model was retained (Morin et al., 2016b; 2017).

**TABLE 3:** Goodness-of-fit of all models.

Model	Chi-square	df	CFI	TLI	RMSEA (90% CI)		RMSEA *p*	SRMR
EFA 1	550.182	27	0.853	0.804	0.115	[0.107, 0.123]	0.000	0.068
EFA 2	337.031	19	0.911	0.831	0.107	[0.097, 0.117]	0.000	0.035
EFA 3	27.708	12	0.996	0.987	0.030	[0.015, 0.045]	0.989	0.012
CFA	180.045	24	0.981	0.971	0.067	[0.058, 0.076]	0.001	0.037
hCFA	225.819	24	0.978	0.967	0.076	[0.067, 0.085]	0.000	0.037
bCFA	163.020	18	0.984	0.968	0.074	[0.064, 0.085]	0.000	0.031
ESEM	26.874	12	0.998	0.994	0.029	[0.014, 0.044]	0.992	0.012
hESEM	26.874	12	0.998	0.994	0.029	[0.014, 0.044]	0.992	0.012
bESEM	0.176	2	1.000	1.003	0.000	[0.000, 0.019]	0.997	0.001

RMSEA *p*: probability that RMSEA is ≤0.05.

#### Research question 1

To determine the extent to which the items of the SEWS exhibited construct-relevant psychometric multidimensionality due to the presence of conceptually related constructs, we compared the CFA to the ESEM model. Overall, both models fit the data well, however, the ESEM model’s goodness-of-fit statistics were marginally better. For example, the CFA exhibited an RMSEA of 0.067, while the ESEM model 0.029, suggesting the ESEM model had less error of approximation and has excellent fit (MacCallum et al., 1996). Latent factor correlations were stronger for the CFA (| *r*| = 0.510–0.808, *M* = 0.652) than the ESEM (| *r*| = 0.428–0.704, *M* = 0.547), suggesting the ESEM model provided a more distinct vantage of the specific factors compared to the CFA. Standardized parameter estimates (factor loadings and residual variances) for both the CFA and the ESEM are presented in Table 4.

**TABLE 4:** Standardized factor loadings and residual variance for the CFA and ESEM.

	ICM-CFA		ESEM			
Items	λ (SE)	δ	λ (SE)			δ
			Ideation	Mechanics	Self-regulation	
**1. Ideation**						
Item 2	0.728 (0.014)**	0.470	0.549 (0.041)**	0.311 (0.034)**	-0.001 (0.034)	0.429
Item 6	0.797 (0.015)**	0.364	0.877 (0.042)**	-0.142 (0.022)**	0.060 (0.031)	0.267
Item 7	0.857 (0.011)**	0.265	0.739 (0.038)**	0.043 (0.032)	0.111 (0.030)**	0.288
**2. Mechanics**						
Item 1	0.838 (0.034)**	0.298	0.190 (0.033)**	0.711 (0.043)**	-0.050 (0.038)	0.363
Item 3	0.717 (0.024)**	0.486	-0.023 (0.039)	0.732 (0.044)**	0.041 (0.031)	0.456
Item 5	0.538 (0.031)**	0.710	-0.106 (0.037)**	0.568 (0.035)**	0.107 (0.040)**	0.680
**3. Self-regulation**						
Item 4	0.805 (0.016)**	0.351	0.157 (0.033)**	-0.003 (0.021)	0.673 (0.034)**	0.376
Item 8	0.724 (0.020)**	0.476	-0.195 (0.024)**	0.007 (0.019)	0.970 (0.035)**	0.282
Item 9	0.800 (0.015)**	0.360	0.221 (0.033)**	0.022 (0.020)	0.576 (0.031)**	0.423

All *a priori* item factor relationships are in grayscale. **p < 0.01.

As expected, an examination of the parameter estimations across both the CFA and ESEM models suggested both models exhibited strong factor to item relations [CFA: | λ| = 0.538–0.857, *M* = 0.756; ESEM (*a priori* items only): | λ| = 0.549–0.970, *M* = 0.711]. As depicted in Table 4, the *a priori* factor loadings across the ESEM model were weaker, suggesting a more accurate depiction of true score variation in comparison to the CFA. Interestingly, target factor loadings across the factors (target only: | λ| = −0.195–0.221, *M* = 0.042) were commonly statistically significant, yet lacked strength. This may indicate that many of the items exhibit a common theme and could better be exhibited by a general factor. Together, these findings suggest the ESEM model more accurately depicted true score variation and accounted for construct-relevant multidimensionality from conceptually related constructs between the latent factors of the SEWS.

#### Research question 2

To examine if the SEWS exhibits construct-relevant psychometric multidimensionality due to the presence of a hierarchically ordered construct, we compared the ESEM model (previously found to be superior to the CFA) to both the hierarchical ESEM and bifactor ESEM models. Drawing from the model selection procedures adopted from Morin et al. (2016b), we did not examine the bCFA. Overall, the fit of all three ESEM models was excellent. Of note, however, the hESEM model fit was asymptotic to that of the ESEM model, as the first-order factor correlations (now disturbances) from the ESEM model were modeled as factor loadings. Because of this, degraded fit, and the fact that second-order models are less interpretable and theoretically useful, this comparison was omitted.

The bESEM model did not converge in its original configuration. In assessing the failed model, it was found that item 1 (“I can write complete sentences”) was heavily negatively skewed, as 80.2% of all responses (*n* = 1,176) were for “*Almost always*.” Taking a substantive approach to this item, it is developmentally appropriate and therefore expected that most secondary students are capable and view themselves as capable of “writing a complete sentence,” and our participants responded accordingly, obviously negatively skewing the response distribution. This item also stands apart from the other two within-factor items that did not reflect a similar response trend. Interestingly, on inspecting the initial confirmatory and base ESEM models, this item did not strongly or abnormally present itself, as the WLSMV is well known to control and handle non-normal item distributions (Finney and DiStefano, 2006). Therefore, identifying this item’s response distribution as problematic only in a bifactor exploratory structural equation scenario is both statistically and pragmatically relevant and useful to future research in this area.

Upon removing this item, the bESEM model adequately converged and a full parameter inspection was conducted to ensure the specific *conventions* factor displayed normal functioning and adequately represented a meaningful latent factor from the two remaining freely estimated items that sufficiently differentiated from the other specific factors and target items (Brown, 2015; Kline, 2016; see Table 5). In doing so, the specific *conventions* factor displayed expected *a priori* and target parameter estimates, clearly delineating a unique and meaningful factor. For this factor alone, *a priori* factor loadings ranged from 0.375 to 0.724, while target (as close to zero as possible) loadings ranged from −0.084 to 0.033 and global factor loadings ranged from 0.326 to 0.474 (see Table 5). Therefore, despite dropping item 1, the bESEM adequately modeled the data well and was used in comparison to the ESEM model.

**TABLE 5:** Standardized factor loadings for bifactor exploratory structural equation modeling solution of the Self-Efficacy for Writing Scale (−se1).

Items	λ (SE)				
	Ideation	Mechanics	Self-regulation	G-factor	δ
**1. Ideation**					
Item 2	0.087 (0.121)	0.154 (0.056)	-0.063 (0.039)	0.723 (0.038)**	0.442
Item 6	0.511 (0.259)*	-0.099 (0.031)**	0.047 (0.021)*	0.750 (0.032)**	0.164
Item 7	0.179 (0.155)	-0.036 (0.033)	0.025 (0.038)	0.820 (0.038)**	0.294
**2. Mechanics**					
Item 3	-0.084 (0.077)	0.375 (0.110)**	-0.045 (0.062)	0.474 (0.046)**	0.625
Item 5	-0.017 (0.069)	0.724 (0.192)**	0.033 (0.026)	0.326 (0.033)**	0.367
**3. Self-regulation**					
Item 4	0.081 (0.047)	-0.013 (0.024)	0.439 (0.036)**	0.654 (0.022)**	0.373
Item 8	-0.025 (0.045)	0.036 (0.021)	0.623 (0.043)**	0.563 (0.032)**	0.294
Item 9	0.009 (0.042)	-0.028 (0.029)	0.336 (0.038)**	0.690 (0.029)**	0.411
ω	0.866	0.654	0.838		
ω_*H*_	0.017	0.039	0.061	0.788	
ω_*HS*_	0.082	0.432	0.292		
% Var. Ind. G-factor	9.46%	65.94%	34.86%		
% Reliable Var.	1.91%	4.31%	6.77%	87.01%	

**p < 0.01, *p < 0.05. All target factors are in grayscale. % Var. Ind. G-factor, percent variation independent of the G-factor; % Reliable Var., percent of reliable variance (ω_*H*_/(1−*total error*)); ω, coefficient omega; ω_*H*_, coefficient omega hierarchical; ω_*HS*_, coefficient omega hierarchical subscale.

Compared to the ESEM model, the bESEM model goodness-of-fit indices were superior (see Table 3). To be clear, however, given the parameter estimation set-up inherent to a bESEM model, it was somewhat expected to find a nearly perfect fit (CFI of 1.0, nearly optimal RMSEA and SRMR, degrees-of-freedom approaching just-identified, and a non-significant Chi-square). Therefore, we inspected the model estimates to best gauge the model’s value over and above the ESEM model.

The bESEM’s G-factor exhibited strong significant factor loadings for all items (| λ| = 0.326–0.820; *M* = 0.625). In most cases, the strength of the factor loading on the G-factor exceeded that of the S-factors. Although factor loading significance is derived from the ratio between the loading strength and its standard error and simply provides a statistical test to determine if the loading is significantly different than zero, it does suggest which loadings likely provide practical significance. For example, although the target loading of item 6 was statistically significant on the *conventions* factor, the strength of the loading suggested it may not be practically significant. Nevertheless, most of the S-factor loadings (| λ| = 0.087–0.724; *M* = 0.409) were markedly stronger than the target loadings (| λ| = −0.009–0.154; *M* = −0.002).

Although the strength of the S-factor loadings are commonly less than that assumed by the G-factor, it can be expected that the factor correlations reported for the ESEM model (| *r*| = 0.428–0.704, *M* = 0.547) were somewhat consumed and re-expressed by increased factor loadings on the G-factor due to having an orthogonal latent factor arrangement. Items 2 and 7 exhibited weak loadings on their *a priori* factor (λ = 0.087 and 0.179, respectively), yet strong loadings on the G-factor (λ = 0.723 and 0.820, respectively), suggesting these items related stronger to global efficacious beliefs toward writing than specific efficacious beliefs toward writing *ideation*. Ultimately, the *ideation* factor appeared to contribute less specific relation within the model (1.91% of the reliable variance) than either the *conventions* or *self-regulation* factors, which exhibit some items that provided stronger parameter estimates toward the S-factor than the G-factor. Additionally, as depicted by OmegaH, the global factor assumed approximately 87% of the reliable variance, suggesting there is a robust theme that runs congruent amongst all the variables therein. Therefore, this model provided a superior depiction of and fit to the data, as suggested by both the goodness-of-fit indices and the extent to which the parameter estimates are generally supportive of a general factor, while also exhibiting specific factor variability. Furthermore, the strength of the G-factor substantiates the need to more accurately model construct-relevant psychometric multidimensionality in relation to globally structured concepts.

Although a well-fitting and interpretable bESEM model was reported, the validity and overall statistical extent to which the latent factors represented each set of items was not explored. Future research would do well to examine more robust statistical approaches to examining if each latent construct was reliable or exhibited construct replicability (Hancock and Mueller, 2001; Rodriguez et al., 2016). Such statistical tests as index H, which is defined as the sum of the ratios of the items’ squared loadings (often explained to be the proportion of variance explained by the factor) on a particular factor to 1 minus the squared loading (unexplained variance), represents a statistical method to examine construct reliability to judge how well a latent variance is represented by the items (Hancock and Mueller, 2001). Additionally, it would be beneficial to examine explained common variance (EVC), which assesses the unidimensionality of the common variance in a set of items to determine if a bifactor representation should actually, given a strong global factor, be treated as unidimensional (Ten Berge and Sočan, 2004; Reise et al., 2013). Future research is needed to fully and statistically establish the appropriateness of a bifactor ESEM representation, as statistical support is essential to ensuring the model is both accepted and appropriate to develop theory and be employed practically. Along this same initiative, future research would do well to also ensure that the ideation factor is statistically meaningful. Using similar tests, research should examine whether this factor can be fully assumed by the global factor.

### Person-centered findings

#### Research question 3

To establish the extent to which the data disaggregates into discernable, meaningful, and interpretable profiles, we first enumerated a calibration data set using the bESEM factor scores. Examining the bESEM calibration enumeration, the non-significant aLMR *p*-value indicated the 3-profile model was favored. The double split-sample cross-validation method, however, suggested the 3-profile solution was not congruent across the entire sample (*p* = 0.0001 and 0.0026, respective to both cross-validation adjusted Chi-square LRTs; see Supplementary material). This split-sample cross-validation method was then deployed to the 4-profile, 5-profile, and 6-profile calibration and validation data, also with no success in replicating the profile configurations across the entire sample.

Despite this, we substantively inspected both the calibration and validation 3-profile solutions and found they had very similar profile means, variances, and proportions. Therefore, we assessed the profile similarity using Morin et al.’s (2016d) multi-group tests of similarity. As evidenced by continued model fit improvements from CAIC, BIC, and aBIC, it was determined that the two samples met *configural, structural, dispersion*, and *distributional* similarity, validating the 3-profile solution across the entire sample. From this, we also statistically and substantively inspected the 4-profile solution to ensure a 3-profile solution provided a better vantage.

The 4-profile solution replicated the major profiles exhibited by the 3-profile solution, but also included a profile that exhibited low *global* and *ideation* (−0.393, −0.653 factor score averages, respectively) averages and a markedly higher (0.653) self-regulation average. However, there was little statistical evidence to select the 4-profile solution over and above the 3-profile solution, as the 4-profile solution was not supported by aLMR *p*-values and the information criteria continue to strongly decline, while the 3-profile model was supported by both a non-significant aLMR *p*-value for the *k* + 1 profile and a notable and obviously elbow plot decline in information criteria (e.g., AIC, BIC, aBIC; Petras and Masyn, 2010; Morin and Marsh, 2015).

Consistent with prior enumeration work and previous recommendations that guide enumeration decisions, a more parsimonious profile solution was retained as the final model, given the statistical support and substantive interpretation (Marsh et al., 2005, 2009).

**FIGURE 2:**
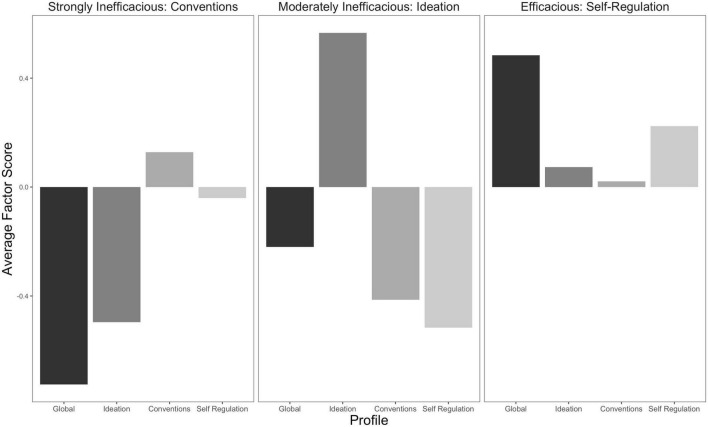
Latent profile model based on bifactor factor scores of the Self-Efficacy for Writing Scale (SEWS).

Table 6 reports each profile’s mean, standard error, and proportions, while Figure 2 depicts this visually. Each profile’s mean latent factor score derived from the bESEM model, and the profile standard error are reported in Table 6. To best describe each profile throughout this study, we named the profiles: profile-1 (Strongly Inefficacious: Conventions), profile-2 (Moderately Inefficacious: Ideation), and profile-3 (Efficacious: Self-Regulation). This naming convention represents the overall general factor valence, while also denoting the strongest positive specific factor. Demographic descriptive statistics are reported in Table 7.

**TABLE 6:** Profile indicator means and standard errors (bESEM).

	Global		Ideation		Conventions		Self-regulation		$\hat{\text{p}}$
Profile	*M*	SE	*M*	SE	*M*	SE	*M*	SE	
1	-0.725	0.077	-0.496	0.049	0.128	0.064	-0.040	0.044	0.267
2	-0.219	0.092	0.566	0.151	-0.414	0.159	-0.516	0.079	0.151
3	0.484	0.112	0.073	0.033	0.021	0.040	0.224	0.098	0.582

$\hat{p}$, proportion of sample. Profile 1: Strongly Inefficacious: Conventions; Profile 2: Moderately Inefficacious: Ideation; Profile 3: Efficacious: Self-Regulation.

**TABLE 7:** Demographic % by profile.

	Profile 1	Profile 2	Profile 3
Total *n* (1,466)	26.67	15.14	58.19
Sex (female)	48.85	48.20	51.70
Minority	46.29	50.45	42.56
8th	10.23	18.02	14.42
9th	36.32	36.49	31.07
10th	53.45	45.50	54.51
ELL	6.39	4.05	2.58
Disability	13.55	15.32	11.96
Gifted	13.04	9.91	16.06

Each percentage represents the percent of each variable represented in each profile. Profile 1: Strongly Inefficacious: Conventions; Profile 2: Moderately Inefficacious: Ideation; Profile 3: Efficacious: Self-Regulation.

The bESEM LPA produced three profiles well-differentiated by *level* differences of global writing self-efficacy. In this case, and relating to the common interpretation of bifactor models, the Strongly Inefficacious: Conventions and Moderately Inefficacious: Ideation profiles exhibited low global writing efficacy yet were well-differentiated through all three of the *specific* factor responses. The *Strongly Inefficacious: Conventions profile*, which included approximately 26% of the participants (*n* = 381), is characterized by the lowest *global* writing self-efficacy, low *ideation*, moderate *conventions*, and relatively average *self-regulation*. Participants in this profile were collectively doubtful, yet exhibited above average confidence for attending to writing *conventions* and much less confidence in their ability to develop and use ideas. Relative to their doubt, students in this profile felt that they could attend to the basic rules of writing such as spelling and punctuation, yet overwhelmingly lacked efficacy about their ability to think of and write about new ideas. Comparatively, the *Moderately Inefficacious* profile portrayed participants who, despite having more than half the low global efficacy, exhibited strong beliefs associated with developing and using ideas, yet were less confident with managing the writing process and employing common writing conventions. As the smallest profile, including approximately 15% of the participants (*n* = 222), the *Moderately Inefficacious: Ideation* profile is also the most obvious in terms of demonstrating the utility of capturing global writing self-efficacy while simultaneously capturing meaningful subscale specificity. Thus, without modeling the collective variability exhibited by all the items, such disparities and unique profiles were, given the demonstration from both the CFA and ESEM LPAs, not likely to be found. *Efficacious: Self-Regulation*, denoted by strong positive global beliefs, average *ideation* and *conventions*, and moderately strong *self-regulation*, is expressed as the normative profile by including almost 60% of participants (*n* = 853). Expressing strong global beliefs, these participants exhibited confidence in all specific facets, especially in their ability to manage the writing process.

#### Research question 4

To assess the concurrent and divergent/discriminant validity of the SEWS, we assessed several predictors and outcomes for their relation to the final enumerated profiles derived from RQ3. First, all demographic predictors were assessed together to provide a more realistic depiction of which variables predicted profile membership, controlling for the other demographic variables. Referencing Table 8, sex, gifted, and disability status were not significant predictors of profile membership. Students from minoritized racial/ethnic backgrounds were reported as being approximately 70% more likely to be in *Moderately Inefficacious: Ideation* than *Efficacious: Self-Regulation*, while ELL students were approximately 300% (or about four times) more likely to be in Strongly Inefficacious: Conventions than *Efficacious*. Results also showed that for each one unit increase in grade level, students had about a 50% greater likelihood of being in Strongly Inefficacious: Conventions relative to *Moderately Inefficacious* and were approximately 35% more likely to be in *Efficacious* when compared to Moderately Inefficacious: Ideation, while controlling for all other demographics.

**TABLE 8:** Predictor coefficients and odds ratios for demographic variables.

Predictors	Profile 1 vs. 3			Profile 2 vs. 3			Profile 1 vs. 2		
	Coefficient	SE	OR	Coefficient	SE	OR	Coefficient	SE	OR
Sex	-0.176	0.173	0.839	-0.188	0.245	0.829	0.013	0.258	1.013
Grade	0.008	0.122	1.008	-0.439**	0.169	0.645	0.446**	0.124	1.562
Minority	0.155	0.144	1.168	0.533**	0.185	1.704	-0.378	0.208	0.685
Gifted	-0.290	0.284	0.748	-0.901	0.471	0.406	0.611	0.491	1.842
Disability	0.225	0.291	1.252	0.354	0.538	1.425	-0.129	0.362	0.879
ELL	1.397**	0.486	4.043	0.906	0.704	2.474	0.491	0.512	1.634

**p < 0.01. Profile 1: Strongly Inefficacious: Conventions; Profile 2: Moderately Inefficacious: Ideation; Profile 3: Efficacious: Self-Regulation.

Next, measurement model (CFA in both cases) factor scores from both the WSES (basic skills factor: ω = 0.89, CI [0.879, 0.902]; advanced skills factor: ω = 0.92, CI [0.911, 0.929]) and the WAS-12 (affect: ω = 0.88, CI [0.867, 0.890]; concern: ω = 0.84, CI [0.828, 0.855]), and first quarter English grades were assessed for their predictive utility toward the likelihood of profile membership. All regression coefficients, standard errors, and odds ratios are reported in Table 9. Outcomes (WSES, WAS-12, and standardized writing assessments) are reported across each profile in Table 10.

**TABLE 9:** Predictor coefficients and odds ratios for WSES and WAS-12 latent factor scores and first quarter English grades.

Predictors	Profile 1 vs. 3			Profile 2 vs. 3			Profile 1 vs. 2		
	Coefficient	SE	OR	Coefficient	SE	OR	Coefficient	SE	OR
WSES – basic	0.365	0.24	1.441	-0.497*	0.207	0.608	0.863**	0.179	2.370
WSES – advanced	-1.719**	0.217	0.179	-0.887**	0.269	0.412	-0.832**	0.189	0.435
WAS12 – affect	-2.168**	0.232	0.114	-1.165**	0.25	0.312	-1.003**	0.188	0.367
WAS12 – concern	1.545**	0.198	4.688	0.983**	0.235	2.672	0.562**	0.183	1.754
Q1 English grades	-0.041**	0.011	0.960	-0.046**	0.011	0.955	0.005	0.006	1.005

*p < 0.05; **p < 0.01. Profile 1: Strongly Inefficacious: Conventions; Profile 2: Moderately Inefficacious: Ideation; Profile 3: Efficacious: Self-Regulation.

**TABLE 10:** Bifactor-ESEM LPA outcomes by profile.

	Profile 1	Profile 2	Profile 3	Summary of significant differences
	***M***	***M***	***M***	
Total *N*	391	222	853	
WSES – basic	−1.014	−1.332	1.077	1 = 2 < 3
*n*	391	222	853	
WSES – advanced	−1.400	−1.303	1.301	1 = 2 < 3
*n*	391	222	853	
WAS-12 – affect	−0.661	−0.209	0.474	1 < 2 < 3
*n*	391	222	853	
WAS-12 – concern	0.496	0.213	−0.376	1 < 2 < 3
*n*	391	222	853	
Grade 8 total performance	446.189	436.446	476.044	2 < 3
*n*	38	38	117	
Grade 8 category 1	34.218	34.279	37.065	1 = 2 = 3
*n*	38	38	117	
Grade 8 category 2	34.770	32.560	37.067	1 > 2 < 3
*n*	38	38	117	
Grade 10 total performance	444.216	431.196	477.077	1 = 2 < 3
*n*	191	93	432	
Grade 10 category 1	35.063	34.236	38.167	1 = 2 < 3
*n*	191	93	432	
Grade 10 category 2	34.594	32.276	38.516	1 > 2 < 3
*n*	191	93	432	

Category 1: research, plan, compose, and revise for a variety of purposes; category 2: edit for correct use of language, capitalization, punctuation, and spelling. Significant differences are *p* < 0.05 from a Wald Chi-square difference test. Total performance, category 1, and category 2 are standardized writing scores. Profile 1: Strongly Inefficacious: Conventions; Profile 2: Moderately Inefficacious: Ideation; Profile 3: Efficacious: Self-Regulation.

## Discussion

The purpose of this study was to examine the multidimensionality of writing self-efficacy using ratings from the adapted SEWS and provide further validity evidence for this measure (Ekholm et al., 2015; Zumbrunn et al., 2016). In summary, the SEWS exhibited evidence of construct-relevant multidimensionality as a product of both latent constructs overlap among the writing SE dimensions (*conventions, self-regulation*, and *ideation*) and the existence of a global writing self-efficacy factor. Using a bifactor ESEM, three latent profiles emerged, characterized by a global indicator across *Strongly Inefficacious, Moderately Inefficacious*, and *Efficacious* themes and specific factor differences between profiles (*Convention, Ideation*, and *Self-Regulation*, respectively). These profiles exhibited strong relationships that aligned with hypothesized expectations.

### RQ1: Conceptual overlap of writing self-efficacy dimensions

Theoretically, Bandura (1997) suggested that multidimensional measures constructed to capture different facets of efficacious beliefs would likely exhibit conceptual overlap. We are aware of no other studies examining if this is truly the case. Our findings suggest that efficacious beliefs are better modeled by an ESEM. Whereas findings across recent writing self-efficacy literature show that efficacious beliefs exhibit latent factor correlations and suggest conceptual overlap (e.g., Bruning et al., 2013; Limpo and Alves, 2017; Ramos-Villagrasa et al., 2018; Zumbrunn et al., 2020, 2021; DeBusk-Lane et al., 2021), the present study provides statistical evidence that such correlations are, in some part, better modeled across all items. Although this is common in the social sciences, especially in psychological measures (see Morin et al., 2016c), it does indicate that there is shared variability across latent factors and, given new statistical approaches (e.g., ESEM), may better be modeled to represent reality more closely.

#### Theoretical implications

The ESEM model reported here provides the current theoretical understanding of writing self-efficacy with important updates. For example, items focused to capture efficacious beliefs of *ideation*, in some part, are also influenced by self-beliefs associated with how well one can perform common writing *conventions*. However, it might be expected that beliefs associated with “…put[ing] my ideas into writing” (item 7) relate to beliefs associated with common writing mechanics such as punctuation, spelling, or forming complete sentences. In this case, as item 7 is phrased, to “put” ideas into writing implies the use and performance of the “generally accepted standards for expressing ideas in writing” (Bruning et al., 2013, p. 28). These cross-concept influences exist for all factors included in this study. Therefore, such cross-concept relations support the notion that efficacious beliefs exist not in extreme *specificity*, but that they prevail broadly in relation to writing. In relation to the adapted SEWS, this suggests that efficacious beliefs associated with the “psychological and linguistic features of the writing process” (Bruning et al., 2013, p. 25) likely exist and can be modeled, in some part, by a global factor, as latent factor correlations remain (| *r*| = 0.428–0.704, *M* = 0.547) despite allowing items to cross-load within the ESEM.

### RQ2: Writing self-efficacy as a hierarchically ordered construct

Using the ESEM model as a basis, we explored the extent to which the SEWS exhibited the presence of a hierarchically ordered global factor. Following Morin et al. (2016a) procedures, the best fitting model from RQ1 was compared to the like (CFA/ESEM) hierarchically- or globally situated model (hESEM/bESEM).

In comparing the ESEM model to the adapted bESEM model, the bESEM model exhibited superior overall goodness-of-fit and anticipated G and S-factor relations. That is, although most (all but one) S-factor *a priori* loadings exhibited stronger loadings for the G-factor, most of the factor loadings continued to provide significant strength over and above the G-factor, while continuing to model minimal target item relations across non-*a priori* item factor relationships. In this case, the continued latent factor correlations found in the ESEM model are re-expressed as the global factor. The *ideation* factor loadings suggest it contributed less to the S-factor than either of the other factors, which exhibited stronger collective loadings to the S-factor. It is important to recall that the G-factor represents the shared variability across all items, while the S-factors express shared variance among the *a priori* items controlling for the G-factor (Reise et al., 2013). These trends are clear in examining the omega coefficients and the percent of variation independent of the G-factor. For instance, for the *ideation* factor, only 9.46% of the reliable variance is independent of the global factor, suggesting the *ideation* factor is almost entirely captured by the global factor. However, despite dropping item 1, the *conventions* factor models 65.94% of the reliable variance after accounting for the global variability, suggesting it is a unique factor (Reise et al., 2013). *Self-regulation* exhibited the second highest amount of variance accounted for independent of the G-factor (34.86%), while also accounting for the highest percent of reliable variability at 6.77%. Therefore, *self-regulation* also appears to be a strong unique factor, as it accounted for a large portion of variability after accounting for the G-factor and models the largest portion of reliable variability after accounting for error. The G-factor, which accounted for 87% of the total reliable variability, suggests that the global factor is ubiquitous across the items and strong.

#### Theoretical implications

The existence and prevalence of such a robust global writing self-efficacy factor extends the theoretical updates provided by the ESEM model. Although efficacy beliefs are commonly understood to be domain-specific (e.g., writing, math, and science) (Bandura, 1986, 1997, 2006, 2018; Pajares, 1996, 2006; Bong and Skaalvik, 2003; Pajares and Usher, 2008; Klassen and Usher, 2010; Usher, 2015; Marsh et al., 2018), our findings suggest there is a strong common theme associated, at least, to the psychological attributes associated with the process of writing. Furthermore, this model also suggests students vary in some of the facets or S-factors. Although students may exhibit collectively high or low efficacious beliefs associated with writing, they still appear to vary between the specific factors. Although this seems logical, as there should be natural S-factor variation at any given point along the (global) continuum of writing beliefs, it may be that such variability is indicative to certain student characteristics, experiences, or methods of writing instruction, as it is well argued that a student’s sociocultural context, or writing community, and collective experiences greatly influence their self-efficacy development (Bandura, 1997; Usher and Pajares, 2008; Graham, 2018; Usher and Weidner, 2018). Additionally, given that the ideation factor was almost entirely modeled by the G-factor may suggest that ideation is instrumental to more macro-level or global efficacy beliefs. As will be discussed in RQ3, profiles derived from this model’s factor scores suggest specific factor ideation to be unique between profiles and may be a strong determinant in differentiating groups of students who globally express less efficacious writing beliefs at-large. It is important to acknowledge, however, that the SEWS was administered within students’ English/Language Arts class and the instructions focused responses on writing conducted in that context. Considering writing efficacy beliefs, and writing beliefs at large, are both a product of prior experience and situated within particular contexts or communities, the degree to which the present model depicts beliefs unique to such is limited (see Graham, 2018). Future research should examine writing in different contexts or communities to inspect potential differences, especially considering the specific factor vantage provided through a b-ESEM.

Nevertheless, this model affords researchers and theorists alike the opportunity to statistically examine a more exact representation of *specific* factor variability over and above a general theme, seemingly providing ample avenues for future research aiming to understanding how various levels of global efficacious beliefs manifest into specific factor expressions and trends (Morin and Marsh, 2015).

### RQ3: Profiles of writing self-efficacy

Once the bESEM model was established as the final model that best depicted the data and best modeled the evident construct-relevant psychometric multidimensionality, we sought to examine how latent factor scores from the final bESEM model disaggregated into interpretable profiles to further establish the measure’s validity. A 3-profile solution was both statistically and substantively superior to model these data.

#### Theoretical implications

The prevalence of profiles differentiated by generalized writing self-efficacy, and the inclusion now of identifiable specific factor differences, informs our current theoretical understanding of how students may exhibit differences in writing self-efficacy. It is important to remember while interpreting the profiles that the specific factors represent variability over-and-above the global factor (Chen et al., 2006). For instance, although profile-1 (*Strongly Inefficacious: Conventions*) exhibits a very low global factor mean, each specific factor mean represents scores derived while accounting for the global factor. For example, in looking at the raw data, two participants that exhibited identical *ideation* factor scores of −1.133 had response patterns of [1, 0, 1] and [2, 1, 1] on the SEWS (for items 2, 6, and 7, respectively), and exhibited global facet factor scores of −1.37 and −0.304, respectively. Although these global factor scores represent the generalization across all 8 items included in the scale, this example clearly demonstrates that the specific factor scores represent important differences not accounted for by the global factor. Bandura’s (1997) contention that more specific beliefs are highly influenced by contextual and experiential factors support our findings; the results here further suggest that these differences are likely expressed differently throughout the continuum of writing self-efficacy. The current findings also suggest that students within profiles might undergo systematic or relatable experiences unique to their writing community (Bandura, 1997; Usher and Pajares, 2008; Graham, 2018). Future research should seek to replicate and further explore such nuances between different contexts and writing communities.

Our findings provide additional theoretical support and evidence that extends writing self-efficacy theory. Bandura (1997) suggests that commonly held or generalized beliefs likely translate into more specifically held facets and these two (generalized and specific beliefs) are inextricably connected. In other words, if a student generally holds less efficacy toward writing, they are also likely to naturally not be very efficacious toward more focused or specific skills associated with writing, such as punctuation or spelling. The present profiles demonstrate this well and support this notion, as both the *Strongly Inefficacious: Conventions* and the M*oderately Inefficacious: Ideation* profiles also exhibited less than average specific factor scores on most specific factors. For example, as a likely product of a strongly globally aligned ideation specific factor, these two profiles are nearly opposite in their expression of ideation beliefs, with the globally positive profile showing more normative specific factor responses. This may suggest that those who hold lower general efficacy beliefs are far more nuanced in their sub-facet beliefs across the specific factors. This is important to both the theoretical understanding of efficacy beliefs and practical efforts of fostering students’ writing efficacy beliefs.

This study further suggests that within this connection or trend between generalized and specific beliefs, there exists rather cohesive groups of students who may exhibit systematic differences among the specific factors. This finding suggests the relationship is not linear within academic domains. Although future research is needed to examine *why* profiles exhibit unique specific factor trends beyond their reported generalization of writing efficacy, we posit that these unique profile trends are produced by differences in students’ interpretations of learning events and in turn, their experiences related to feelings of self-efficacy. Results from DeBusk-Lane et al.’s (2021) support this notion, finding differences in not just the sources reported between profile, but the specific occasions or interpretations of sources they reported, it is likely that students who exhibit generally less (or more) efficacious beliefs of their writing ability interpret and develop their beliefs from disparate sources.

To date, only one known study has been published and employed a bESEM LPA on self-efficacy data. Work by Perera et al. (2019) examined teacher efficacy profiles derived from a bESEM model. Although they state no major theoretical implications to the self-efficacy literature, their profiles resemble and exhibit similar *level* and *shape* effects as reported here. Findings from both Perera et al.’s (2019) and the present study support that writing self-efficacy is best modeled as a general global factor with more specific self-efficacy dimensions (*conventions, self-regulation*, and *ideation*).

### RQ4: Validity evidence for writing self-efficacy

Our findings align well with the literature that suggests writing efficacy beliefs and writing motivation in general tends to decline through the secondary school years (Pajares and Valiante, 1999; Pajares et al., 2007a; Pajares and Usher, 2008; Usher and Pajares, 2008; Klassen and Usher, 2010), although the probability of membership into Strongly Inefficacious: Conventions vs. *Moderately Inefficacious* is an interesting point with the stark differences between *ideation*. Because some students also exhibited higher probabilities of being in Strongly *Efficacious*, relative to *Moderately Inefficacious*, by grade, perhaps this indicates beliefs – and instructional contexts – diverge to some degree throughout these years of schooling. Indeed, students’ likelihood to become more aware of their own domain-specific abilities in comparison with their peers during the middle school years is well documented in the literature (e.g., Eccles et al., 1993; Eccles and Roeser, 2009; Wigfield et al., 2015). Caution should be taken, however, as these data are cross-sectional and longitudinal inferences should not be taken.

#### Theoretical implications

In terms of predictors of profile membership, the primary contribution is that these predictions replicate prior findings throughout literature and further substantiate the theoretical understanding of how personal factors relate to expressed efficacious beliefs (Bandura, 2008; Pajares and Usher, 2008). Interestingly, the lack of statistical significance for sex, which has been a focal point in writing efficacy research (Pajares et al., 1999, 2007a; Pajares and Valiante, 1999, 2001; Villalón et al., 2015; De Smedt et al., 2017), is, perhaps, the most surprising finding amongst the predictors. Our results however align with recent findings from DeBusk-Lane et al.’s (2021), who also found that sex was not predictive of profile membership when accounting for other demographic variables. Despite this incongruence across the literature highlights that more research is needed to further unpack how sex – and, though not explored directly in this work, gender (see Pajares et al., 2007b) – relates to efficacy beliefs.

Like DeBusk-Lane et al. (2021), we also found strong statistically significant predictive effects associated with differences in grade-level. In both cases, those in higher grades were more likely to be in a less efficacious profile. However, the present findings indicate a stronger relationship of those in higher grades being predicted to be members of the Strongly *Efficacious* profile, suggesting that students become more differentiated as they progress through these grades. This could be explained by developmental changes in efficacious beliefs that are strongly influenced by everchanging, dynamic, and normative experiences that all mix with, inevitably, rapidly developing biological influences (Bandura, 1997). Additionally, it is likely that students (grades 8–10) transitioning to high-school also tend to become more academically specialized. It would be expected that those who ascribe to and focus on more non-writing domains become less efficacious in their writing and account for some students of higher grades having a higher likelihood of membership in less efficacious profiles.

Aside from student demographics, we also assessed the predictive nature of those who identified as gifted, having a disability, or being an English language learner. Surprisingly, neither those identified as being gifted or having a disability were significantly predictive of profile membership (García and de Caso, 2004; García and Fidalgo, 2008). English language learners’ identity, however, was significantly predictive of profile membership such that these students had a higher likelihood of being members of the *Strongly Inefficacious: Conventions* profile, as compared to the *Efficacious: Self-Regulation* profile. Given prior literature in these areas, though limited, these trends align and would be expected (Teng et al., 2018).

To further provide validity evidence, we also examined the predictive value of both the WSES and the WAS-12 on profile membership. Interestingly, both measures were highly predictive across all profiles. Those with higher WSES’s basic skills were more likely to be in Strongly Inefficacious: Conventions compared to *Moderately Inefficacious*, yet also more likely to be in *Efficacious: Self-Regulation* than *Moderately Inefficacious* profiles. This, along with grade differences, may suggest that as students gain more writing skills, they also become more efficacious and comfortable with, at least in regard to the Strongly Inefficacious: Conventions *profile*, writing *conventions*. Comparatively, those with higher WSES advanced skills were generally more likely to be in profiles with higher efficacy beliefs. This is to be expected, as the crosswalk between basic and advanced skills as operationalized by the WSES appears to translate well to the SEWS’ *conventions* and *ideation* factors, respectively. So, in this case, it is logical for those with stronger writing skills and beliefs to be more associated with membership profiles exhibiting stronger efficacy beliefs. Nevertheless, those with higher skills scores were approximately 82% more likely to be in the Efficacious: Self-Regulation profile, relative to the Strongly Inefficacious: Conventions *profile*. Diverging results of the WAS-12’s affect (liking) and concern (writing anxiety) indicated that those who reported liking writing more exhibited stronger and significant predictions into more positive profiles (3 > 2 > 1), in-line with research between anxiety and writing self-efficacy (Pajares and Johnson, 1994; Pajares and Valiante, 1999; Goodman and Cirka, 2009; Martinez et al., 2011; Sanders-Reio et al., 2014; Limpo, 2018).

We also assessed how the profiles related to both the WSES and the WAS-12. Both factors of the WSES aligned with the global factor indicator in each profile. Participants reported less efficacy in less globally efficacious profiles. Interestingly, however, Strongly Inefficacious: Conventions (profile-1) and Moderately Inefficacious: Ideation (profile-2) exhibited similar averages for both the basic and advanced factors (although basic was reported less efficacy for profile-2 than 1). Responses to the WAS-12’s affect (liking) writing factor were in-line with our hypotheses, such that those with a stronger sense of efficacy toward writing exhibited a stronger affliction toward writing. Conversely, those who reported less efficacy toward writing (members of lower profiles), exhibited a stronger relation to the concern factor of the WAS-12. These findings provided validity evidence that the profiles are aligned to the well-established positive relationship between writing self-efficacy and writing affect, and the negative relationship between writing self-efficacy and writing apprehension. Furthermore, our results provide new insights into the relationship between writing self-efficacy and apprehension. Although apprehension aligns with lower efficacy beliefs, the profiles identified allow a better understanding of how specific factors associated with the writing process differentially relate. That is, apprehension may play a large part in shaping a student’s beliefs around creativity and ideation, yet have little impact on their beliefs around conventions because such skills – and related efficacy beliefs – are more durable or reinforced. Future research would do well to inspect these interactions to better understand how specific efficacy beliefs interact with apprehension, especially in students who hold lower writing efficacy beliefs.

As would be expected, first quarter English grades significantly predicted membership into efficaciously stronger profiles, however, no predictive relationship was found that differentiated between the Strongly Inefficacious: Conventions or *Moderately Inefficacious* profiles. These results provide both concurrent and divergent validity evidence of both the profiles and the adapted SEWS’ global indicator (Pajares and Johnson, 1994; Pajares and Valiante, 1999; Goodman and Cirka, 2009; Martinez et al., 2011; Sanders-Reio et al., 2014; Limpo, 2018), yet also suggest grades may not well differentiate between those who hold lower efficacy beliefs in general. Future research is needed to further explore the relationship between students’ grades and the experiences that generate such grades, and how students’ experiences with writing shape their writing efficacy beliefs.

Additionally, a standardized writing assessment was used to establish predictive validity of the profiles. Grade 8 total standardized writing scores mimicked earlier findings that have tended to find clear and statistically significant differences between *Efficacious: Self-Regulation* above that of both Strongly Inefficacious: Conventions and *Moderately Inefficacious*. Although no clear differences were found among grade 8’s category 1 scores, category 2 scores indicated that Strongly Inefficacious: Conventions (which exhibited above average efficacious beliefs associated with writing *conventions*) was significantly higher than *Moderately Inefficacious* (which exhibited less than average *conventions*). Considering category 2 primarily involved editing for “…punctuation, and spelling,” it is no surprise that those who exhibited stronger beliefs also performed better in this area. Grade 10 scores were reported in a similar manner across all three standardized test scores, also finding that category 2 was higher for those who exhibited above average *conventions*. In this case, using the bESEM model likely attenuated these differences and demonstrated the advantage of more accurately and precisely capturing specific factor differences among the profiles. As such, our findings imply that relations between writing self-efficacy and both grades’ standardized writing scores may be more related to specific factor differences than generalized efficacy beliefs. This would make practical sense, as the standardized tests used in this study focused on specific writing processes, such as editing. These results highlight how standardized tests may not fully tap into all aspects of the writing process and may not differentially relate to students of varying levels of generalized efficacy beliefs associated with writing. This suggests that when inspecting the relationship between grades and efficacy beliefs it is especially important to ensure skill alignment between both performance and beliefs. This line of reasoning is not meant to negate the differences between the *Efficacious: Self-Regulation* profile and the two lowest profiles, but that there were either no discernable differences between Strongly Inefficacious: Conventions and *Moderately Inefficacious*, or that Strongly Inefficacious: Conventions exhibited stronger standardized category 2 scores than *Moderately Inefficacious*, despite *Moderately Inefficacious* reporting stronger global efficacy. Although the predictive nature of the bESEM model was not assessed, the standardized test outcomes reported between profiles here may offer important clues as to the nature of such a prediction. Given writing self-efficacy has been positively associated with writing performance (see Pajares, 2003; Pajares et al., 2007a), the present study adds further evidence that there is a clear difference present between profiles with higher and lower efficacy beliefs and the state-wide standardized writing scores. Furthermore, our findings also offer theoretical support for how scales should be developed. Criterial alignment (*correspondence*), whereby the measure aligns with the performance outcome, often results in greater performance prediction (Pajares, 1996; Bandura, 1997, 2006; Klassen and Usher, 2010; Marsh et al., 2018). In this case, higher *conventions* scores related to the performance outcome of the standardized test’s category 2 outcome, which measured a student’s ability to edit. Future research should continue to assess the relational and predictive association between each profile and important outcomes such as grades, with the ultimate intent to focus improvement.

## Implications for educators

The present study may offer important information for educators about the development of students’ writing self-efficacy. As the findings demonstrate, students who exhibit strong writing efficacy beliefs, or even those who appear doubtful, may also substantively differ on the extent to which they hold efficacious beliefs of their ability to attend to the rules of writing (*conventions)*, their ability to develop and use ideas (ideation), or their ability to self-manage throughout the writing process (self-regulation). Understanding these trends in the classroom may offer benefits in terms of targeting opportunities for students to develop mastery experiences (Pajares, 1996, 2003; Villalón et al., 2015), while also acknowledging that students’ efficacy beliefs may largely be held more generally toward writing. Our findings showing that a rather substantial group of students who commonly view writing with less confidence simultaneously hold much less efficacious beliefs in relation to using and crafting ideas, suggests educators may do well to focus on creating, molding, developing, and employing ideas during writing tasks (more so than focus on writing conventions or self-regulation).

Despite the statistical and theoretical value of determining which indicators from the bESEM model best predict meaningful and important outcomes, others have noted that improving writing self-efficacy should be “advanced as an explicit goal for writing instruction” (Usher and Pajares, 2008; Bruning and Kauffman, 2015, p. 160). This suggests that there is great value in cultivating writing self-efficacy in general. As such, the present findings, which depict groups of students differentiated by a collective and global sense of efficacious beliefs toward writing, support the notion that efforts to foster stronger efficacy beliefs across all areas of ideation, conventions, and self-regulation may enhance students’ writing performance. This is not meant to denounce the present study’s findings, but to clearly articulate that the robust presence of a global factor (that represents ∼87% of the reliable variation) and the meaningful presence of the specific factors may suggest viable instructional pathways both globally and in a targeted sense that require future research to fully examine.

## Conclusion

The present study demonstrated that a strong general factor exists among all the items of the SEWS, while the specific factors (i.e., *conventions, self-regulation*, and *ideation*) continue to be well-represented. This suggests that writing self-efficacy simultaneously exists along both a collective spectrum of efficacious beliefs and is expressed differentially among the original multidimensional factors of the SEWS. Participants, when grouped into three profiles (Strongly Inefficacious: Conventions, *Moderately Inefficacious*, and *Efficacious: Self-Regulation*), differentiated by global factor shape and exhibited unique differences along the specific factors. Generally, these profiles were well differentiated by global efficacious beliefs, while specific factor differences were mainly seen between the two lower efficacious profiles (Strongly Inefficacious: Conventions, *Moderately Inefficacious: Ideation*) across all three specific factors. Both student grade level and racial/ethnic minority status were predictive of profile membership, while the WSES and WAS-12 also demonstrated concurrent and divergent validity across the profiles. Further, the profiles were also validated using grades, WSES, and the WAS-12 as outcomes to provide concurrent and discriminant validity evidence. Together, these findings provide evidence that the adapted SEWS contains construct-relevant psychometric multidimensionality as a product of both conceptual overlap between the specific factors and the existence of a global or generalized theme congruent to all items, therefore suggesting the often used, and perhaps over-used, CFA depiction is less than optimal. These findings extend the current theoretical understanding of writing self-efficacy in terms of the hierarchical, multidimensional structure of this complex construct, how writing self-efficacy manifests across unique student profiles, and how student characteristics and learning outcomes relate to membership in one of the three profiles.

## Data availability statement

Requests to access these datasets should be directed to the corresponding author. Due to participant privacy and the provided ethnical consent, limited data access should be expected.

## Ethics statement

The studies involving human participants were reviewed and approved by the Human Research Protection Program/Institutional Review Board, Virginia Commonwealth University. Written informed consent from the participants’ legal guardian/next of kin was not required to participate in this study in accordance with the national legislation and the institutional requirements.

## Author contributions

MD-L and SZ conceived the project and designed the study. MD-L wrote the manuscript. SZ, CB, MB, RB, and AS provided the feedback and edits. All authors made substantial contributions and reviewed and approved the completed manuscript.
