# Supplementary material for: Variable- and person-centered approaches to examining construct-relevant multidimensionality in writing self-efficacy
Source: Front Psychol. 2023 Feb 20;14:1091894. doi: 10.3389/fpsyg.2023.1091894 (PMC9986581; doi:10.3389/fpsyg.2023.1091894)
Supplement: Supplementary file 1 [file Data_Sheet_1.PDF]

Supplementary Materials

Adapted Self-Efficacy for Writing Scale (SEWS; Ekholm et al., 2015; Zumbrunn et al., 2016)

We now would like you to think about writing in your English/Language Arts Class. For each statement, please choose the world that best describes you.

1. I can write complete sentences.
2. I can think of many words to describe my ideas.
3. I can punctuate my sentences correctly.
4. I can concentrate on my writing for a long time.
5. I can spell my words correctly.
6. I can think of many ideas for my writing.
7. I can put my ideas into writing.
8. I can avoid distraction when I write.
9. I can keep writing even when it is difficult.

Response Scale: 4 point scale: 1 = *Almost never*, 2 = *Sometimes*, 3 = *Often*, 4 = *Almost Always*

LPA Enumeration Fit Indices for CFA and bESEM Calibration Data

Model	*N*	Parameters	Loglikelihood	cf	AIC	CAIC	BIC	aBIC	Entropy	aLMR	aLMR *p-value*
1-Calibrate CFA LPA	734	6	-2309.580	0.934	4631.161	4631.277	4658.752	4639.700			
2-Calibrate CFA LPA	734	10	-1869.684	1.333	3759.367	3759.671	3805.353	3773.599	0.796	847.677	0.000
3-Calibrate CFA LPA	734	14	-1621.817	1.366	3271.634	3272.218	3336.013	3291.558	0.852	477.637	0.000
**4-Calibrate CFA LPA**	**734**	**18**	**-1493.890**	**1.374**	**3023.780**	**3024.737**	**3106.553**	**3049.397**	**0.851**	**246.515**	**0.012**
5-Calibrate CFA LPA	734	22	-1430.506	2.683	2905.011	2906.434	3006.178	2936.321	0.866	122.141	0.744
6-Calibrate CFA LPA	734	26	-1374.602	2.545	2801.205	2803.191	2920.766	2838.207	0.870	107.725	0.512
7-Calibrate CFA LPA	734	30	-1332.163	1.131	2724.326	2726.972	2862.281	2767.021	0.869	81.781	0.016
4-Validate (fixed) CFA LPA	732	0	-1458.138		2916.276	2916.276	2916.276	2916.276	0.848		
4-Validate (free) CFA LPA	732	18	-1442.008	1.468	2920.015	2920.974	3002.739	2945.583	0.855		
4-Calibrate (fixed) CFA LPA	734	0	-1511.973		3023.945	3023.945	3023.945	3023.945	0.854		
1-Calibrate ESEM LPA	734	6	-2789.029	0.937	5590.058	5590.174	5617.649	5598.597			
2-Calibrate ESEM LPA	734	10	-2455.822	1.288	4931.644	4931.948	4977.629	4945.875	0.744	642.087	0.000
3-Calibrate ESEM LPA	734	14	-2273.916	1.317	4575.832	4576.416	4640.211	4595.757	0.821	350.531	0.001
**4-Calibrate ESEM LPA**	**734**	**18**	**-2186.542**	**1.208**	**4409.084**	**4410.041**	**4491.857**	**4434.701**	**0.815**	**168.369**	**0.004**
5-Calibrate ESEM LPA	734	22	-2150.404	1.606	4344.808	4346.231	4445.975	4376.118	0.810	69.637	0.502
6-Calibrate ESEM LPA	734	26	-2122.752	1.091	4297.503	4299.489	4417.065	4334.506	0.836	53.286	0.001
7-Calibrate ESEM LPA	734	30	-2107.389	1.064	4274.777	4277.423	4412.733	4317.473	0.819	29.604	0.014
4-Validative (fixed) ESEM LPA	732	0	-2204.554		4409.107	4409.107	4409.107	4409.107	0.804		
4-Validative (free) ESEM LPA	732	18	-2184.242	1.241	4404.484	4405.443	4487.208	4430.052	0.784		
4-Calibrate (fixed) ESEM LPA	734	0	-2206.448		4412.896	4412.896	4412.896	4412.896	0.785		
1-Calibrate Alt bESEM LPA	734	8	-3037.983	0.981	6091.966	6092.165	6128.754	6103.352			
2-Calibrate Alt bESEM LPA	734	13	-2996.172	1.056	6018.345	6018.851	6078.125	6036.846	0.483	81.161	0.000
**3-Calibrate Alt bESEM LPA**	**734**	**18**	**-2938.864**	**1.226**	**5913.728**	**5914.685**	**5996.501**	**5939.345**	**0.643**	**111.245**	**0.006**
4-Calibrate Alt bESEM LPA	734	23	-2916.516	1.434	5879.032	5880.587	5984.798	5911.765	0.624	43.381	0.433
5-Calibrate Alt bESEM LPA	734	28	-2891.042	1.197	5838.084	5840.388	5966.842	5877.933	0.715	49.450	0.042

6-Calibrate Alt bESEM LPA	734	33	-2859.280	1.180	5784.561	5787.767	5936.312	5831.526	0.777	61.654	0.043
7-Calibrate Alt bESEM LPA	734	38	-2807.559	1.293	5691.118	5695.383	5865.862	5745.199	0.840	47.243	0.106
3-Validate Alt (free) bESEM LPA	732	18	-2918.269	1.103	5872.538	5873.497	5955.262	5898.106	0.575		
3-Validate Alt (fixed) bESEM LPA	732	0	-2945.291		5890.581	5890.581	5890.581	5890.581	0.639		
3-Calibrate Alt (fixed) bESEM LPA	734	0	-2962.970		5925.939	5925.939	5925.939	5925.939	0.553		
4-Validate Alt (fixed) bESEM LPA	732	0	-2922.352		5844.704	5844.704	5844.704	5844.704	0.616		
4-Validate Alt (free) bESEM LPA	732	23	-2890.026	1.095	5826.052	5827.611322	5931.755	5858.722	0.626		
4-Calibrate Alt (fixed) bESEM LPA	734	0	-2947.739		5895.478	5895.478	5895.478	5895.478	0.610		
5-Validate Alt (free) bESEM LPA	732	28	-2854.074	1.162	5764.147	5766.457	5892.829	5803.920	0.744		
5-Validate Alt (fixed) bESEM LPA	732	0	-2912.297		5824.594	5824.594	5824.594	5824.594	0.711		
5-Calibrate Alt (fixed) bESEM LPA	734	0	-2941.111		5882.223	5882.223	5882.223	5882.223	0.724		
6-Validate Alt (fixed) bESEM LPA	732	0	-2884.852		5769.705	5769.705	5769.705	5769.705	0.778		
6-Validate Alt (free) bESEM LPA	732	33	-2794.400	1.250	5654.800	5658.015	5806.461	5701.675	0.845		
6-Calibrate Alt (fixed) bESEM LPA	734	0	-2869.077		5738.155	5738.155	5738.155	5738.155	0.839		
bESEM Multi-Group Configural	1466	37	-6873.285	1.305	13820.571	13822.540	14016.311	13898.774	0.761		
bESEM Multi-Group Structural	1466	25	-6885.670	1.466	13821.341	13822.244	13953.598	13874.181	0.742		
bESEM Multi-Group Dispersional	1466	21	-6885.998	1.579	13813.995	13814.635	13925.091	13858.381	0.743		
bESEM Multi-Group Distributional	1466	19	-6887.129	1.601	13812.259	13812.785	13912.774	13852.417	0.744		
**Final bESEM LPA - Full Sample**	**1466**	**18**	**-5870.977**	**1.649**	**11777.954**	**11778.43**	**11873.179**	**11815.999**	**0.583**		

Chi-square Loglikelihood Ratio Tests

CFA								
4-Profile Validation Fixed w/ Calibration svalues Compared to Validative Freely Estimated								
L0	L1	c0	c1	p0	p1	cd	TRd	*p*-value
-1458.1380	-1442.0080	0.0000	1.4675	0.0000	18.0000	1.4675	21.9830	0.2327
4-Profile Calibration Fixed w/ Validative svalues Compard to Calibrated Freely Estimated								
L0	L1	c0	c1	p0	p1	cd	TRd	*p*-value
-1511.9730	-1493.8900	0.0000	1.3737	0.0000	18.0000	1.3737	26.3274	0.0925
ESEM								
4-Profile Validation Fixed w/ Calibration svalues Compared to Validative Freely Estimated								
L0	L1	c0	c1	p0	p1	cd	TRd	*p*-value
-2204.5540	-2184.2420	0.0000	1.2407	0.0000	18.0000	1.2407	32.7428	0.0179
4-Profile Calibration Fixed w/ Validative svalues Compard to Calibrated Freely Estimated								
L0	L1	c0	c1	p0	p1	cd	TRd	*p*-value
-2206.4480	-2186.5420	0.0000	1.2083	0.0000	18.0000	1.2083	32.9488	0.0169
Bifactor ESEM								
3-Profile Validation Fixed w/ Calibration svalues Compared to Validative Freely Estimated								
L0	L1	c0	c1	p0	p1	cd	TRd	*p*-value
-2945.2910	-2918.2690	0.0000	1.1028	0.0000	18.0000	1.1028	49.0062	0.0001
3-Profile Calibration Fixed w/ Validative svalues Compard to Calibrated Freely Estimated								
L0	L1	c0	c1	p0	p1	cd	TRd	*p*-value
-2962.9700	-2938.8640	0.0000	1.2262	0.0000	18.0000	1.2262	39.3182	0.0026
4-Profile Validation Fixed w/ Calibration svalues Compared to Validative Freely Estimated								
L0	L1	c0	c1	p0	p1	cd	TRd	*p*-value
-2922.3520	-2890.0260	0.0000	1.0951	0.0000	23.0000	1.0951	59.0375	0.0001
4-Profile Calibration Fixed w/ Validative svalues Compard to Calibrated Freely Estimated								
L0	L1	c0	c1	p0	p1	cd	TRd	*p*-value
-2947.7390	-2916.5160	0.0000	1.4335	0.0000	23.0000	1.4335	43.5619	0.0059
5-Profile Validation Fixed w/ Calibration svalues Compared to Validative Freely Estimated								
L0	L1	c0	c1	p0	p1	cd	TRd	*p*-value
-2947.7390	-2854.0740	0.0000	1.1624	0.0000	28.0000	1.1624	161.1579	0.0000
5-Profile Calibration Fixed w/ Validative svalues Compard to Calibrated Freely Estimated								
L0	L1	c0	c1	p0	p1	cd	TRd	*p*-value
-2941.1110	-2891.0420	0.0000	1.1967	0.0000	28.0000	1.1967	83.6784	0.0000
6-Profile Validation Fixed w/ Calibration svalues Compared to Validative Freely Estimated								
L0	L1	c0	c1	p0	p1	cd	TRd	*p*-value
-2884.8520	-2794.4000	0.0000	1.2496	0.0000	33.0000	1.2496	144.7695	0.0000
6-Profile Calibration Fixed w/ Validative svalues Compard to Calibrated Freely Estimated								
L0	L1	c0	c1	p0	p1	cd	TRd	*p*-value
-2869.0770	-2859.2800	0.0000	1.1796	0.0000	33.0000	1.1796	16.6107	0.9922

Note. L0 and L1 = Logliklihood values; c0 and c1 = MLR scaling correction factor; p0 and p1 = Parameters; cd = scaling correction; TRd = Chi-square difference test.

[State] Quality Criterion Tool for Performance-Based Assessment

This document is designed to help guide the development of performance-based assessments in [the state] by establishing common language and important criteria for performance-based assessments in K-12 schools. It is not a "how to" guide and there will be additional templates, processes, and professional development provided to utilize the tool across grades and content areas. It is also an important guide for shifting teachers' instructional methods and assessment practices that will engage students in meaningful work in order for them to develop the knowledge, skills, and dispositions to be college and work ready.

<table>
<tr><th colspan="2"></th></tr>
<tr><th>Generic Criteria</th><th>[State] Quality Criteria for Performance-Based Assessment (PBA)</th></tr>
<tr><td>(1) Standards/ Intended Learning Outcomes</td><td>PBAs are aligned to content standards (including essential knowledge and skills) and other intended learning outcomes (such as dispositions or behaviors) of the curriculum.
PBAs:

● Align to a developmentally appropriate, cogent (i.e., complex, coherent, important) sets of intended learning outcomes;
● Occur within the scope of the grade-level curriculum and within the vertical sequence of the subject;
● Build toward an accurate, deep, understanding of content, processes, and skills; and
● May integrate intended learning outcomes from two or more subject areas.
● May align to students' development as critical thinkers, effective communicators, constructive collaborators, creative thinkers, and/or contributing citizens
● May integrate technology-related competencies, if appropriate to the intended learning outcomes and/or response format of a PBA.

Note: PBAs aim to develop deeper learning in students, which may be defined as a set of six interrelated competencies: mastering rigorous academic content, learning how to think critically and solve problems, working collaboratively, communicating effectively, directing one's own learning, and developing an academic mindset — a belief in one's ability to grow.</td></tr>
</table>

(2) Cognitive Demand	PBAs require students to engage in higher order thinking over some extended period of time (whether several minutes of multiple weeks). Higher order thinking is characterized by:<br><br>• More than simple recall<br>• Conceptual understanding, application, analysis, evaluation, synthesis, or original creation<br>• Subject-specific competencies such as scientific inquiry, historical analysis, persuasive writing, or mathematical reasoning.
(3) Authenticity	PBAs engage students in relevant tasks that are representative of the real world and/or relevant to the discipline. Authenticity may be relevant to jobs/careers, future responsibilities as adults, current interests of students as young people, or academic tasks relevant to the discipline. The authenticity of PBAs is often characterized by a/an:<br><br>• Engaging scenario<br>• Realistic role<br>• Relevant task<br>• Target audience<br>• Meaningful product or outcome<br>• Genuine constraints<br>• Relevant expectations<br><br>Authenticity can be accentuated through demonstration of learning within a novel situation, connections to the students' community/world, students' understanding of the purpose of the task, and student choice of task, process, and/or response format (i.e., product or performance).
(4) Verbal Reasoning / Explanation	PBAs support language development and verbal reasoning connected to the discipline, such as constructive conversations with peers.<br>PBAs should include student explanations with supporting details/rationale, in writing or other multimodal format.

(5) Success Criteria	PBAs include accurate, reasonably objective criteria by which to judge students' performance relative to expectations. A set of success criteria typically takes the form of a/an:<br><br>• Analytic rubric<br>• Holistic rubric<br>• Rating scale<br>• Checklist of Quality Indicators<br><br>Success criteria strengthen the potential reliability and validity of PBAs by improving the likelihood of consistently applied expectations (reliability) of the intended content and skills (validity), rather than focusing on the surface features of a product or performance.<br>Given the central role of language usage in PBAs, success criteria may include the purposeful and effective conveyance of ideas through language regardless of subject-area being assessed.<br>Results can be used to demonstrate adequate academic progress in a subject and to inform instructional decisions.<br>Success criteria—especially when written in student-friendly language—can provide opportunities for self-assessment, peer and teacher/expert feedback, reflection, and revision, all of which are examples of *assessment for learning*.
(6) Student Directions / Prompt	The task prompt is clear, developmentally appropriate, and is aligned to criterion being assessed (validity).The task is free of biased language, stereotypes, and/or sensitive, offensive, or inappropriate topics.
(7) Accessibility	The performance assessment accommodates the participation of all students. It allows students to access the assessment through multiple entry points, while providing diverse ways of responding to the task to support accessibility. Directions for teachers for a given PBA can define appropriate, allowable supports or alternatives to facilitate accessibility while maintaining the validity and reliability of the PBA.

(8) Feasibility	Formats of PBAs may vary based on features such as:<br><br>● Duration (e.g., a few minutes, a class period, multiple days, or weeks)<br>● Number of intended learning outcomes to be demonstrated<br>● Degree of student choice in process and/or response format<br>● Degree of expected teacher direction or support during completion<br><br>PBAs may take the form of constructed-response items, stand-alone assessments, curriculum-embedded assessments, or extended projects. Regardless of the form, PBAs must be feasible to implement with respect to time, materials, and space available.	
(9) Instruction	PBAs necessitate instructional approaches in the classroom and learning experiences for students that lead to students' deeper conceptual understandings and mastery of subject-specific skills.	Each school board shall annually certify that it has provided instruction and administered an alternative assessment, consistent with Board guidelines, to students in grades three through eight in each Standards of Learning subject area in which a Standards of Learning assessment was not administered during the school year.

(10) Teacher Materials / Substantiation	PBAs provide accurate, complete information and clear directions to teachers to help ensure some fidelity of administration and use. PBAs may be substantiated by:<br><br>● a copy of the PBA itself,<br>● an assessment blueprint,<br>● a scoring protocol, sample responses,<br>● and/or training materials for teachers.<br><br>To strengthen the potential validity and reliability of PBAs, they should be designed and developed using steps before (e.g., template, table of specifications), during (e.g., teacher directions for administration of the PBA), and after use (e.g., inter-rater reliability check).	The design, development, administration, substantiation, and use of LAAs should emphasize collaborative effort among teachers and administrators.
